# Chaikhwa Nani Nelson: Overcoming Cancer and Leading Oncology in Botswana

**DOI:** 10.7759/cureus.68506

**Published:** 2024-09-03

**Authors:** Nandan M Shanbhag, Sneha N Shanbhag

**Affiliations:** 1 Internal Medicine, College of Medicine and Health Sciences, United Arab Emirates University, Al Ain, ARE; 2 Radiation Oncology/Palliative Care, Tawam Hospital, Al Ain, ARE; 3 Internal Medicine, Dubai Physiotherapy and Rehabilitation Centre, Dubai, ARE

**Keywords:** historical review, chevening scholarship, cancer support, osteosarcoma, oncology nursing, chaikhwa nani nelson, cancer, botswana, medical innovators, historical vigenette

## Abstract

Chaikhwa Nani Nelson, born on February 11, 1992, in Tutume village, Botswana, is a remarkable figure in oncology nursing who has transformed personal adversity into a driving force for change in cancer care. Diagnosed with osteosarcoma in 2013, she faced the life-altering challenge of undergoing a lower limb amputation and enduring the rigors of chemotherapy. Despite these hardships, Chaikhwa's resolve to support others battling cancer only strengthened. Her journey led her to become an oncology nurse, where she tirelessly advocates for patient-centered care and the need for compassionate healthcare professionals. Chaikhwa's efforts have been recognized internationally, with accolades including the Mandela Washington Fellowship, the Queen's Young Leader Award, and a prestigious Chevening Scholarship, through which she earned her MSc in Clinical Oncology. She founded Botswana's first cancer support group, providing much-needed resources and community for patients and their families. Chaikhwa Nani Nelson's story is a testament to resilience and dedication, making her a pioneering leader in oncology care in Botswana.

## Introduction and background

Chaikhwa Nani Nelson, born on February 11, 1992, in Tutume village, Botswana, stands as a beacon of hope and resilience in the field of oncology nursing [1]. Her journey from a small village in Botswana to becoming an influential leader in cancer care is marked by determination, courage, and an unwavering commitment to improving the lives of others. Diagnosed with osteosarcoma at the age of 21 years, Chaikhwa faced the daunting challenges of dealing with cancer and living with a disability post-amputation. Despite these life-altering experiences, today, Chaikhwa is a celebrated oncology nurse, an advocate for cancer patients, and a trailblazer in the healthcare community of Botswana. Her story is a powerful example of how personal adversity can be harnessed to drive significant, positive change in society.

Chaikhwa Nani Nelson's early life in Tutume village laid the foundation for her enduring spirit and commitment to service. After completing her primary and secondary education in her hometown, she pursued a qualification in General Nursing at the Institute of Health Sciences, setting the stage for her career in healthcare. In 2013, shortly after beginning her career as a registered nurse, Chaikhwa was diagnosed with osteosarcoma, a rare and aggressive form of bone cancer [2]. The diagnosis led to the amputation of her leg, a life-altering event that could have ended her career in nursing. However, Chaikhwa’s determination to continue serving others saw her return to the field with renewed purpose. She shifted her focus to oncology, recognizing the critical need for compassionate and empathetic care for cancer patients, a realization that was deepened by her own experiences during treatment.

Chaikhwa's professional journey has been marked by significant achievements and recognition, including the Mandela Washington Fellowship, the Queen's Young Leader Award, and the Chevening Scholarship, through which she completed a Master of Science in Clinical Oncology at the University of Birmingham. Beyond her personal, academic, and professional achievements, Chaikhwa's most profound impact has been on her community. She founded Botswana’s first cancer support group, providing a vital network of resources and emotional support for patients and their families. Her work has not only improved the lives of those she directly cares for but has also raised awareness about cancer and the importance of early detection and support systems in Botswana.

Chaikhwa's story is one of triumph over adversity, driven by a deep-seated commitment to making a difference in the lives of others. Her journey from patient to caregiver, from a small village to international recognition, encapsulates the power of resilience and the impact of compassionate care in the field of oncology.

## Review

Early life and education

**Figure 1:**
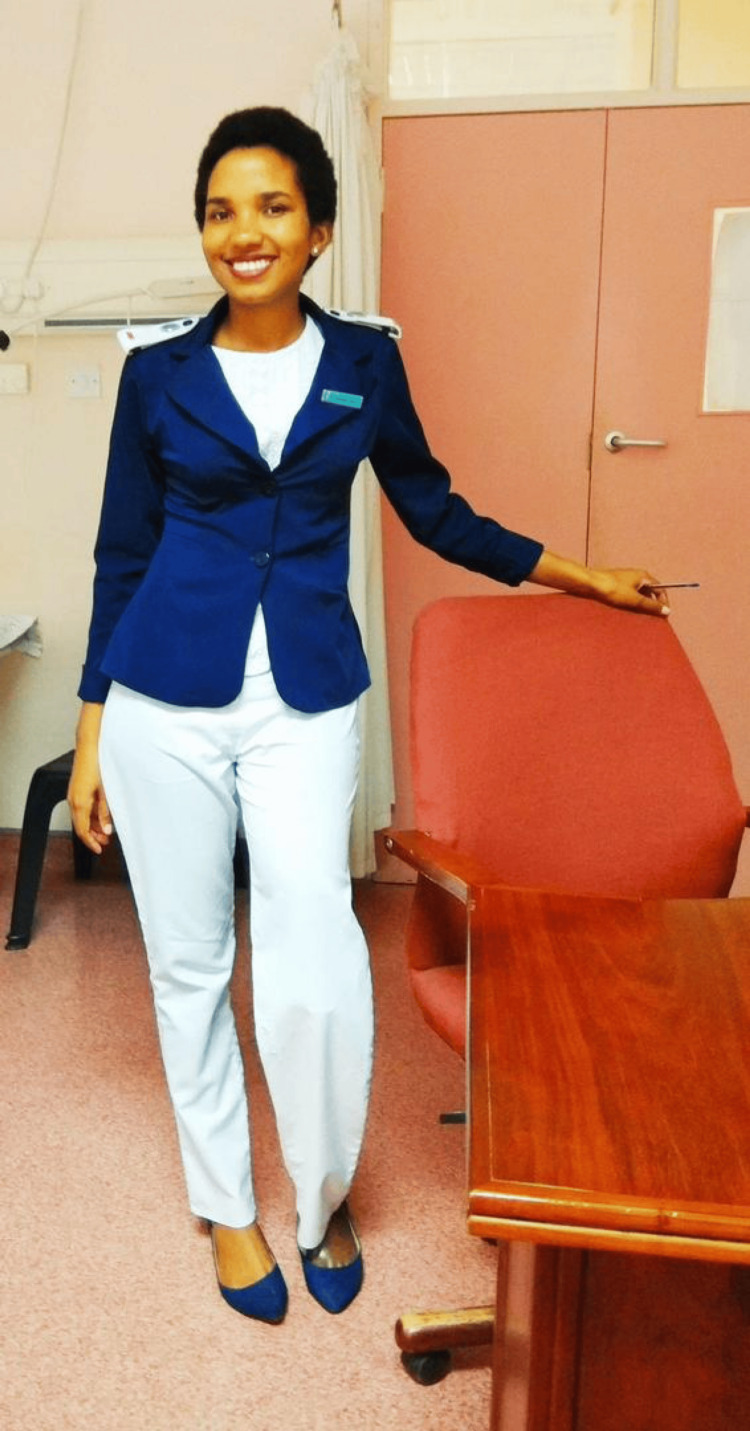
Chaikhwa Nani Nelson The image has been provided by Ms. Nelson and used with written permission.

Chaikhwa Nani Nelson was born on February 11, 1992, in the small village of Tutume, located in the Central District of Botswana. Growing up in a rural area, Chaikhwa was nurtured by a close-knit community that instilled in her the values of resilience, compassion, and service. Her early education in Tutume laid the foundation for her future career in nursing, as she showed an early interest in healthcare and helping others. After completing her secondary education, she pursued a General Nursing qualification at the Institute of Health Sciences, driven by a desire to contribute to the well-being of her community. This initial training equipped her with the skills and knowledge needed to begin her career as a registered nurse in 2013 (Figure 1).

Diagnosis and battle with osteosarcoma

**Figure 2:**
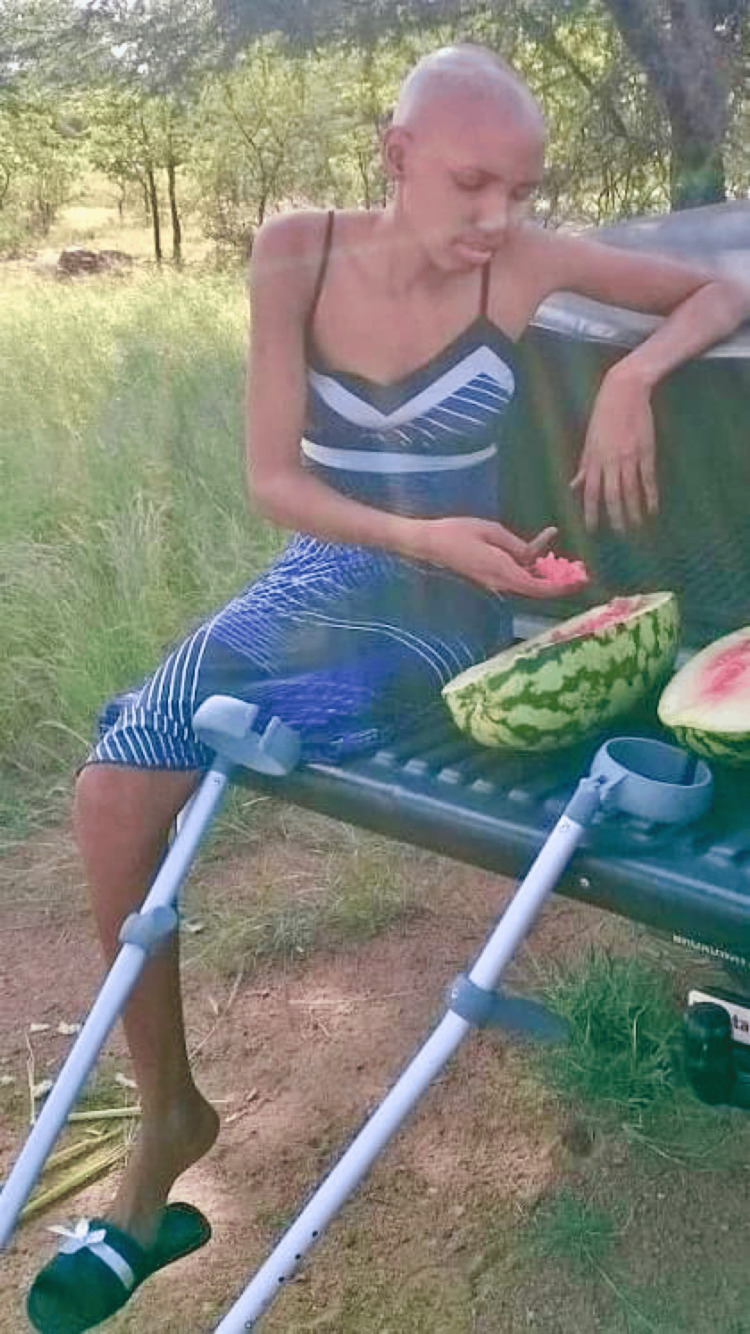
Chaikhwa during chemotherapy (2014) The image has been provided by Ms. Nelson and used with written permission.

In November 2013, just months after starting her career as a nurse, Chaikhwa's life took a dramatic turn when she was diagnosed with osteosarcoma, a rare form of bone cancer. The diagnosis was devastating, requiring her to undergo a leg amputation within a month. The amputation, while life-saving, marked the beginning of a new and challenging chapter in her life. Chaikhwa reflected on the profound change, stating, “After 21 years of total physical ability, I had to adjust to living with a disability.” Her battle with cancer extended beyond surgery. Beginning chemotherapy in January 2014, Chaikhwa encountered the harsh realities of cancer treatment, including severe side effects that tested her physical and emotional limits (Figure 2).

Despite the challenges, she faced her treatment with unwavering determination, holding onto the belief that "I am me, and what happened to someone else will not happen to me." Her resilience during this period was bolstered by the steadfast support of her family, friends, and colleagues.

Transition to oncology and advocacy

Her personal battle with cancer opened Chaikhwa's eyes to the significant gaps in support for cancer patients in Botswana. Botswana, like many other countries in sub-Saharan Africa, faces significant challenges in providing comprehensive cancer care to its population. The burden of cancer in Botswana has been steadily increasing, partly due to the rise in life expectancy and the adoption of more Westernized lifestyles, which have contributed to an increase in the incidence of non-communicable diseases, including cancer [3,4]. One of the major challenges in Botswana is the limited availability of specialized cancer care services. Historically, cancer diagnosis and treatment options were sparse, with most patients having to travel abroad for advanced care [5]. However, in recent years, the government of Botswana has made strides in improving cancer care infrastructure within the country. This includes the establishment of oncology departments in major hospitals and the expansion of training programs for healthcare professionals [6,7]. Despite these improvements, access to cancer care in Botswana remains uneven, particularly in rural areas where healthcare facilities are limited. There is also a significant shortage of oncologists and specialized nurses, which hampers the ability to provide timely and effective treatment [8]. Furthermore, the stigma associated with cancer and the limited awareness about the disease contribute to late-stage diagnoses, which negatively impacts treatment outcomes [9]. Ms. Nelson observed that many patients lacked the necessary emotional and practical support to navigate their treatment journey [10]. This realization sparked a passion in Chaikhwa to make a difference in the lives of other cancer patients. She requested a transfer to the Oncology department, where she could directly contribute to the care of individuals facing the same challenges she had overcome.

**Figure 3:**
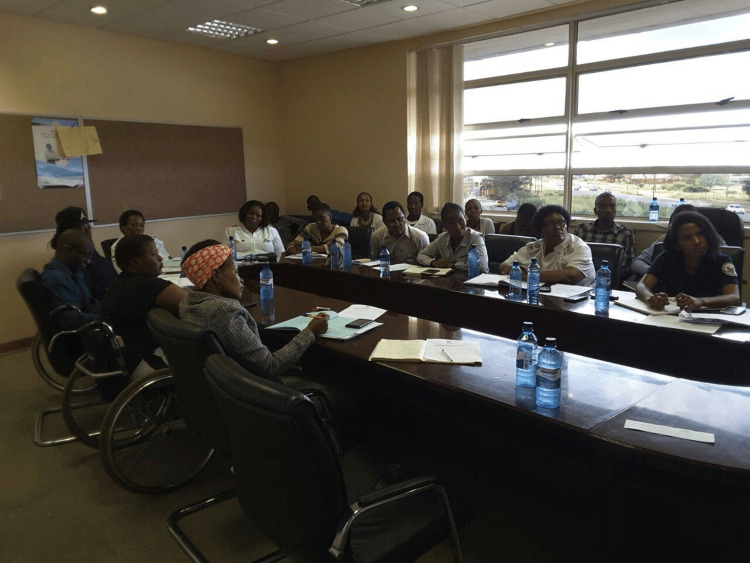
Cancer support group in Botswana The above picture is available for the public on Ms. Chaikhwa Nani Nelson's Facebook page and produced here with permission.

Chaikhwa’s transition to oncology was driven by a deep understanding of the needs of cancer patients. She knew firsthand the importance of having healthcare professionals who are not only skilled but also compassionate and empathetic. In response to the challenges, initiatives such as the Botswana National Cancer Registry were established to collect data on cancer incidence and outcomes, which is crucial for planning and improving cancer care services. Additionally, partnerships with international organizations and neighboring countries have been instrumental in providing training, resources, and support to Botswana's healthcare system. Her work in oncology became more than just a job; it was a mission to fill the void she had seen during her own treatment. Chaikhwa’s approach to patient care is guided by a philosophy of empathy, ensuring that every patient feels seen, heard, and supported. The establishment of cancer support groups, such as the one founded by Chaikhwa Nani Nelson in Serowe, represents a significant advancement in addressing the psychosocial needs of cancer patients in Botswana. These support groups provide a platform for patients and their families to share experiences, receive emotional support, and access information about cancer care (Figure 3).

Awards and recognition

**Figure 4:**
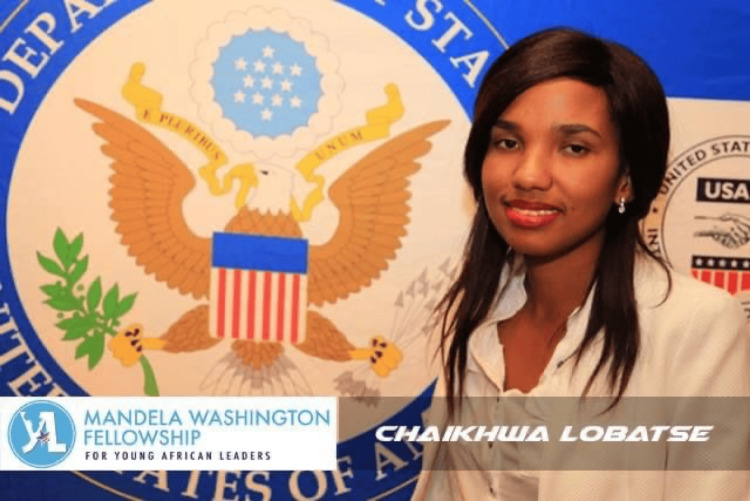
Mandela Washington Fellowship (2016) This image, originally found on the U.S. Embassy Gaborone's Facebook page in the post "Meet our LUCKY 26 - Mandela Washington Fellowship 2016," was accessed on August 15, 2024 [11]. The image is used with permission from Chaikwa Nelson, who retains all rights to the photograph. There is no copyright infringement as the necessary permissions have been obtained.

Chaikhwa’s efforts in the field of oncology have not gone unnoticed. Her work has garnered international recognition, beginning with the Mandela Washington Fellowship in 2016 (Figure 4) [11]. This prestigious fellowship allowed her to study leadership at Georgia State University in the US, where she expanded her knowledge of policy-making and leadership. The experience also deepened her passion for palliative care, as she visited nursing homes and hospices, gaining insights into the care of the dying.

**Figure 5:**
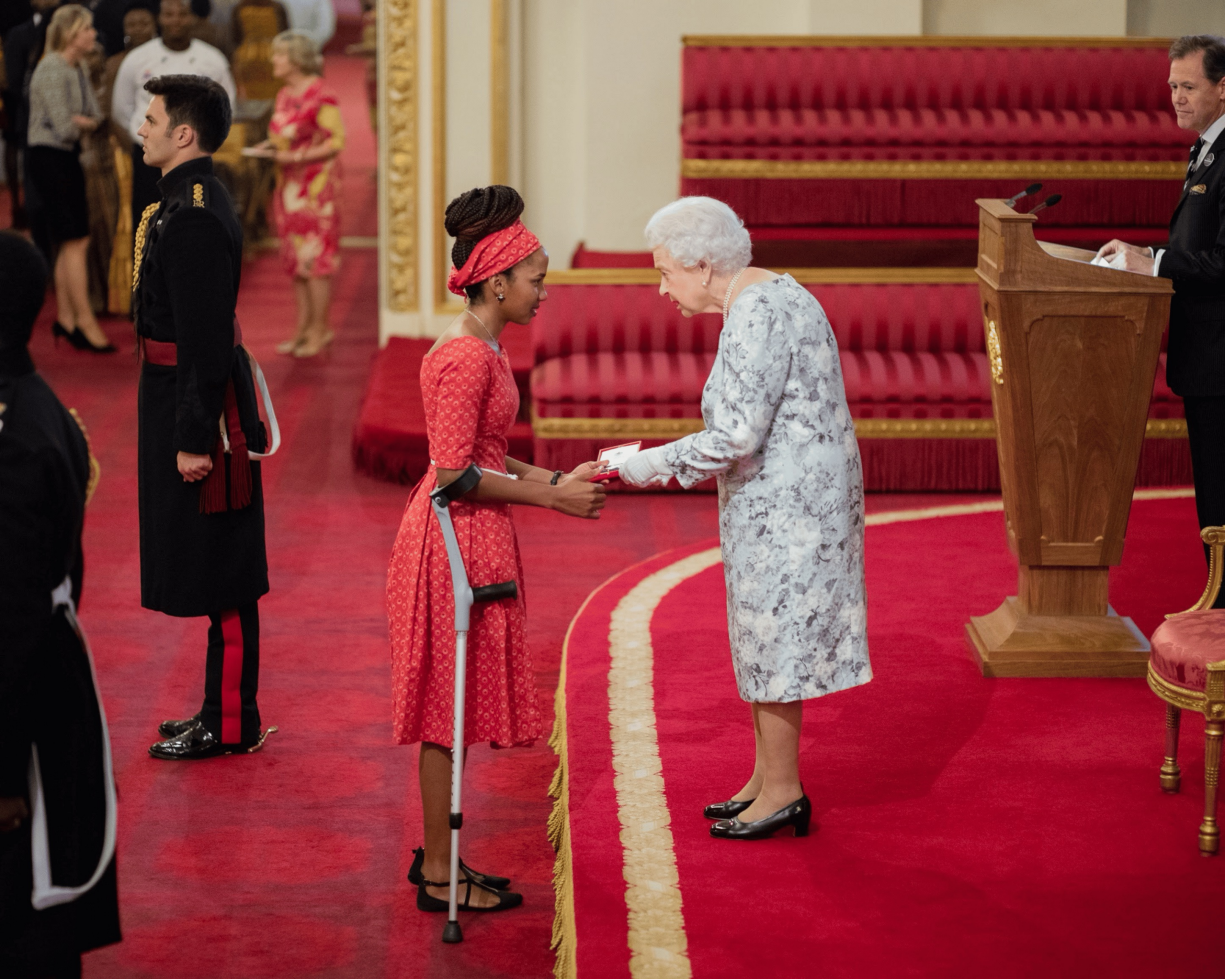
Queen's Young Leader Award (2017) Chaikhwa Nani Nelson receiving the prestigious Queen's Young Leader Award from Her Majesty The Queen in 2017. As the only Motswana and one of the few Africans honored that year, this recognition celebrated her exceptional contributions to promoting health and well-being in Botswana. This image, originally found on the Queen's Young Leaders website in the profile of Chaikhwa Lobatse, was accessed on August 15, 2024 [12]. The image is used with permission from Chaikhwa Nelson, who retains all rights to the photograph. There is no copyright infringement as the necessary permissions have been obtained.

In 2017, Chaikhwa was honored with the Queen’s Young Leader Award, making her the only Motswana recipient that year. She traveled to the United Kingdom to receive the award from Her Majesty the Queen, a testament to her dedicated work in promoting health and well-being in Botswana (Figure 5) [12]. The award included a free online course from the University of Cambridge, further enhancing her leadership skills.

**Figure 6:**
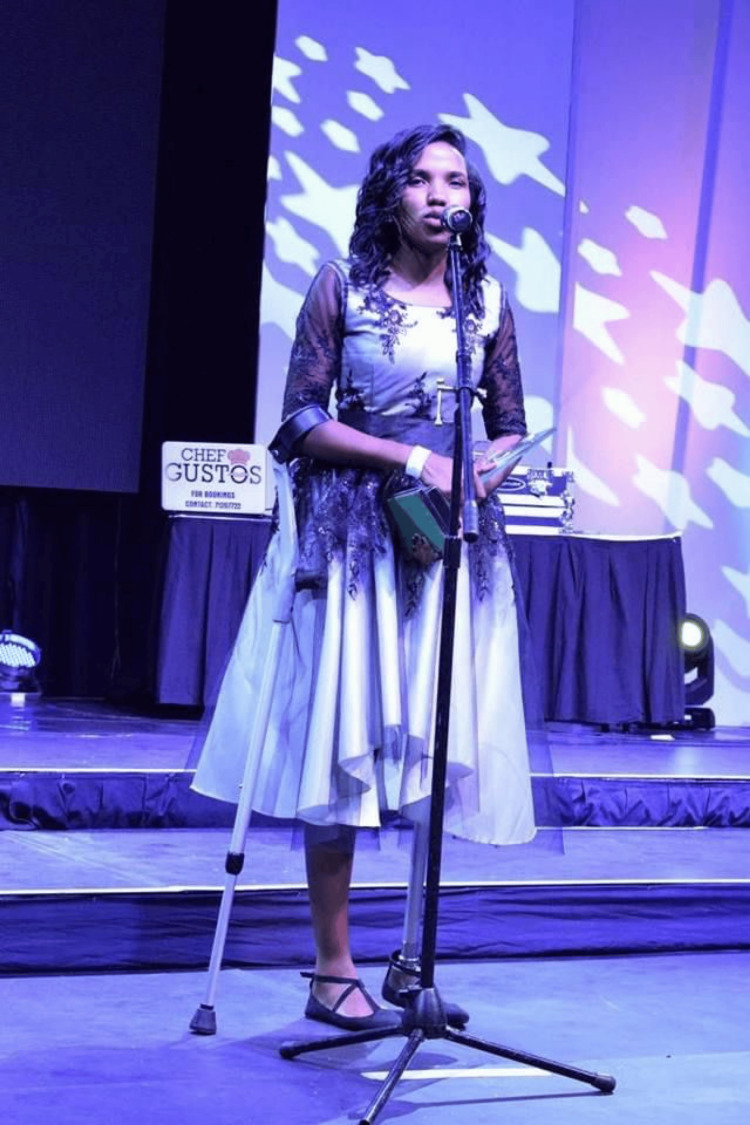
Botswana’s best youth promoting health and wellbeing (2017) This image, originally found on the Botswana Leaders' Facebook page in the post "Best Youth Promoting Health and Wellness - Congratulations for the Award," was accessed on August 15, 2024 [13]. The image is used with permission from Chaikhwa Nelson, who retains all rights to the photograph. There is no copyright infringement as the necessary permissions have been obtained.

That same year, she was also recognized as Botswana’s Best Youth Promoting Health and Wellbeing, an accolade that highlighted her influence as a young leader in healthcare (Figure 6) [13].

**Figure 7:**
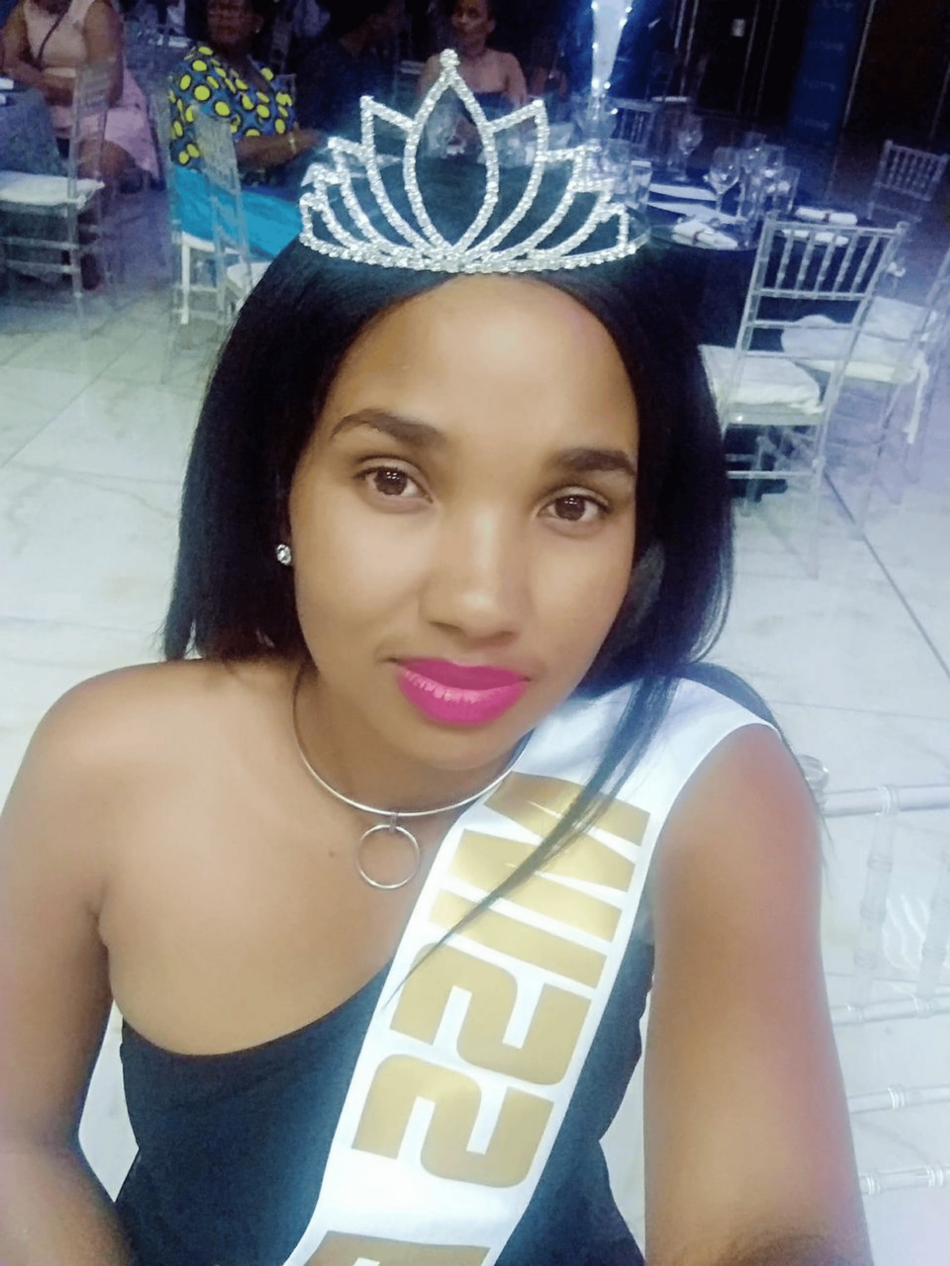
Miss Botswana Nurses Day (2018) In 2018, Chaikhwa Nani Nelson was crowned Miss Botswana Nurses Day, a title that recognized her outstanding contributions to the nursing profession. The photograph is provided by Chaikhwa Nelson, who retains all the rights and has reproduced it here with written consent.

In 2018, her peers in the nursing community crowned her Miss Botswana Nurses Day, celebrating her contributions to the profession (Figure 7).

Academic pursuits and the Chevening Scholarship

**Figure 8:**
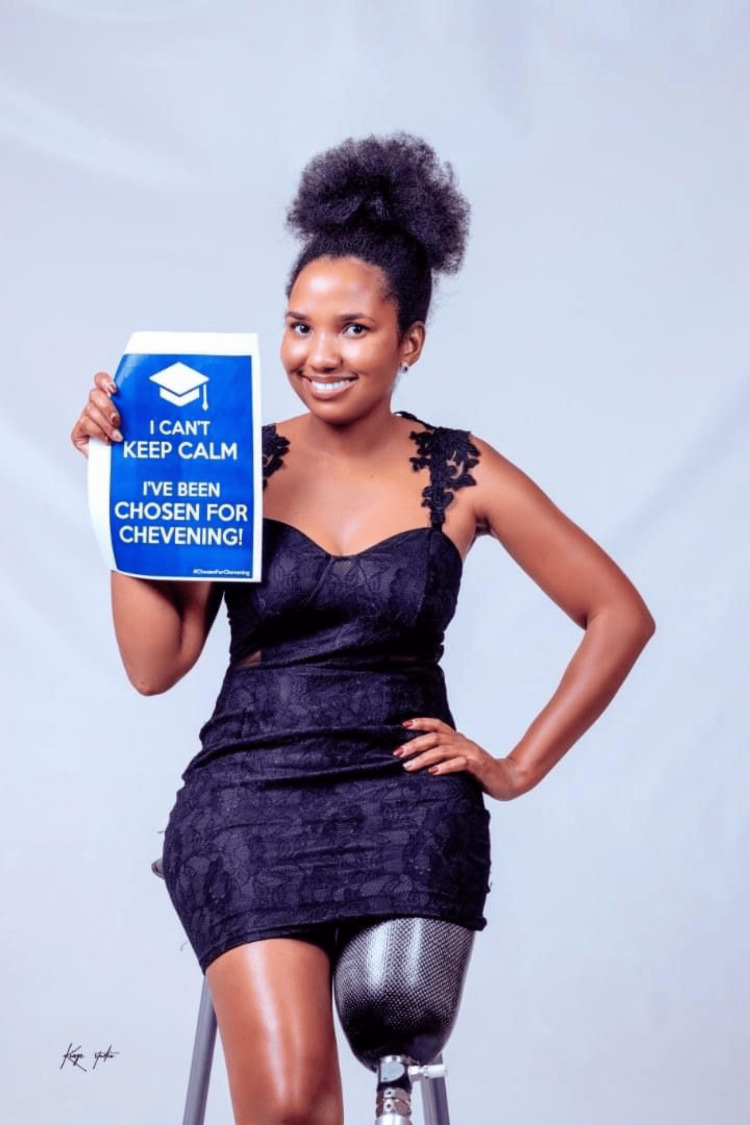
Chevening Fellowship (2018) The photograph is provided by Chaikhwa Nelson, who retains all the rights, and reproduced here with written consent.

**Figure 9:**
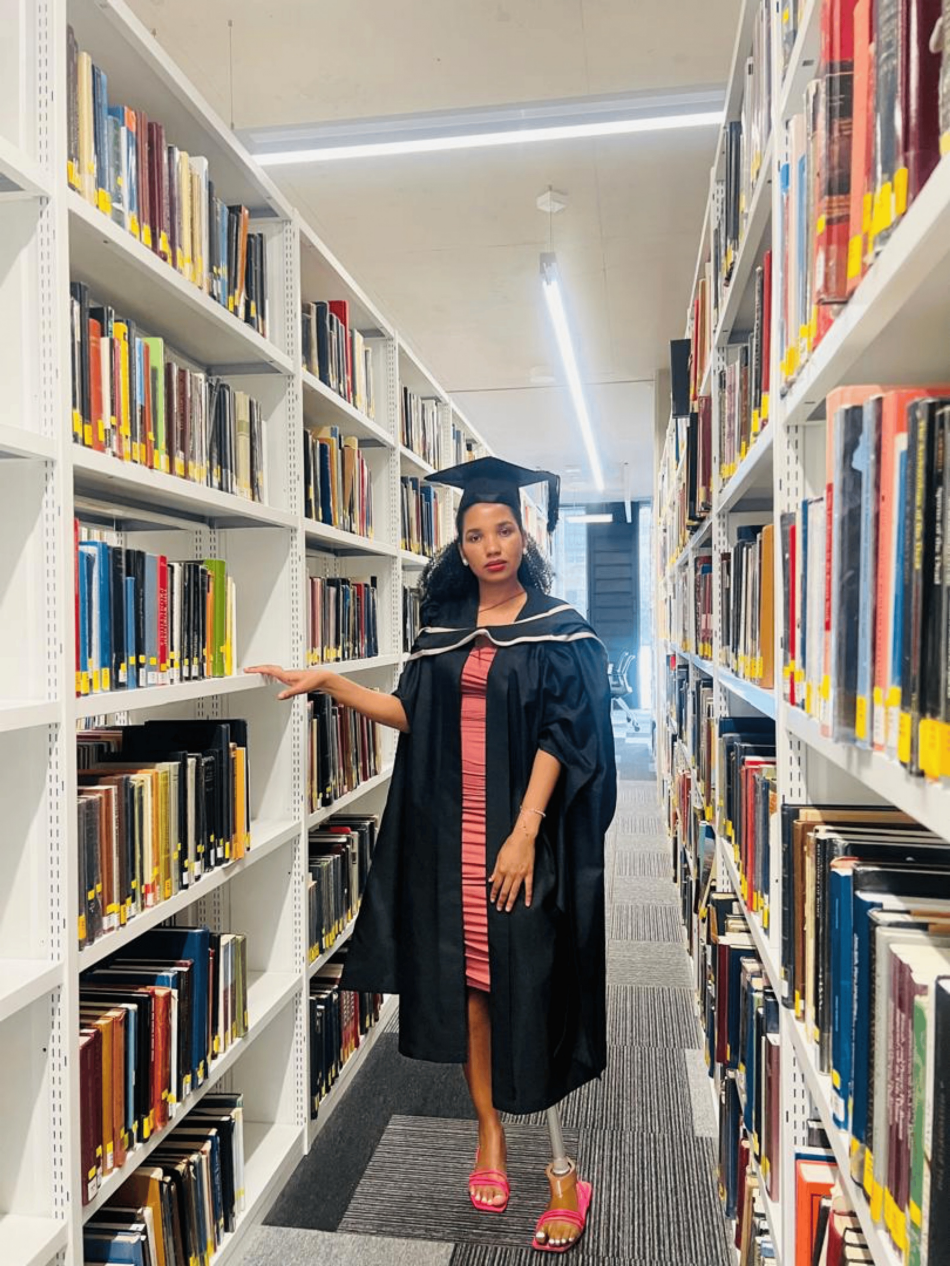
University of Birmingham The picture provided by Ms. Nelson depicts the library at the University of Birmingham while she was pursuing her Master’s in Clinical Oncology. The photograph is provided by Chaikhwa Nelson, who retains all the rights, and reproduced here with written consent.

**Figure 10:**
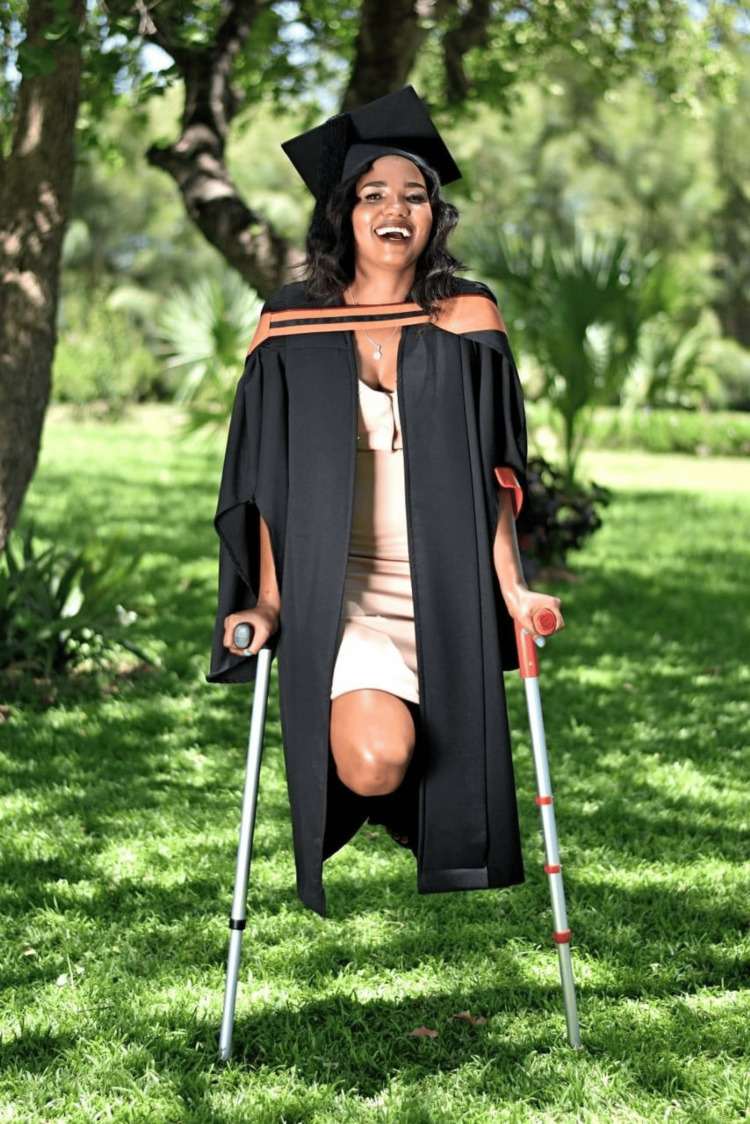
Graduation ceremony Master’s in Clinical Oncology In 2018, she became the first nurse in Botswana to attain a Master's in Clinical Oncology. The photograph is provided by Chaikhwa Nelson, who retains all the rights, and reproduced here with written consent.

In 2021, Chaikhwa was awarded the prestigious Chevening Scholarship, an opportunity that allowed her to further her education in the United Kingdom. She chose to pursue a Master of Science in Clinical Oncology at the University of Birmingham, a decision that reflected her commitment to advancing cancer care in Botswana. The Chevening Scholarship is awarded to individuals who have demonstrated leadership potential and a commitment to their communities, both of which Chaikhwa exemplifies (Figures 8-10).

Her studies in Clinical Oncology provided her with advanced knowledge and skills that she has since applied to her work in Botswana. Upon her return, she continued to be a strong advocate for cancer patients, using her expertise to improve the quality of care and support available to them.

Establishing Botswana’s first cancer support group

One of Chaikhwa’s most significant contributions to cancer care in Botswana has been the establishment of the country’s first cancer support group in Serowe. This group focuses on raising awareness about cancer, promoting healthy living, and providing much-needed support to those fighting cancer and their caregivers. The group serves as a critical resource for patients, offering emotional support, practical advice, and a sense of community during their treatment journey.

Through this support group, Chaikhwa has been able to address some of the gaps she observed during her own treatment. The group has become a lifeline for many patients, helping them to navigate the complexities of cancer treatment and recovery. Chaikhwa’s leadership in this area has been instrumental in improving the overall experience of cancer patients in Botswana.

Palliative care clinic

**Figure 11:**
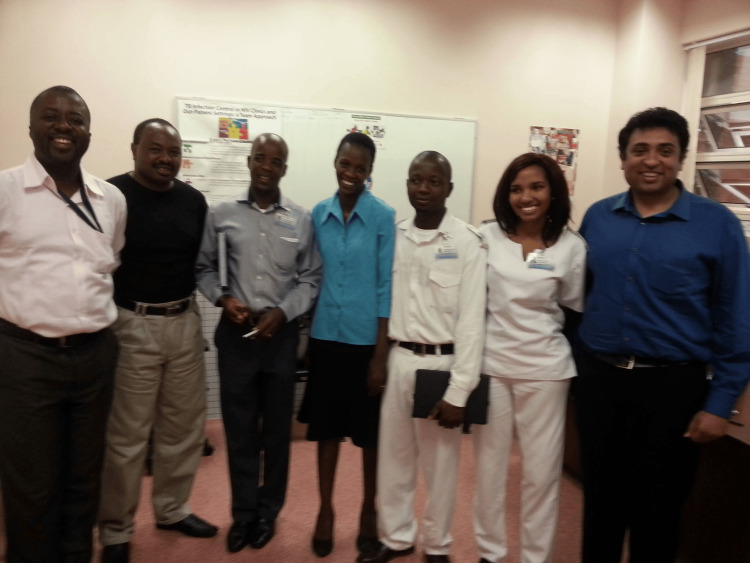
Multidisciplinary team From left: Dr. Patrick Manshimba (Medical Officer), Mr. Terence Zitha (Pastor), Mr. Gabakopane (Social Worker), Ms. Oganeditse Supang (Dietitian), Mr. Aatamela Makgaola (Registered Nurse), Chaikhwa Lobatse-Nelson (Oncology Nurse), and Dr. Nandan M. Shanbhag (Chair, Department of Oncology). This photograph captures the multidisciplinary team at Sekgoma Memorial Hospital in Serowe, Botswana, who played a pivotal role in establishing and advancing palliative care services in the region. The photograph was captured on the first author’s device and did not infringe upon any copyrights. Reproduced with permission.

In 2016, the team established Botswana’s first palliative care clinic, pioneering the integration of a multidisciplinary team to provide comprehensive care. This team included an oncologist, general physician, pastor, nurse, social worker, dietitian, physiotherapist, occupational therapist, psychologist, family medicine nurse, and nurse coordinator. The team held weekly hour-long meetings with patients and their families to discuss challenges in detail and collaboratively develop solutions. These meetings were centered around advanced care planning, ensuring that all aspects of the patient's and family's needs were addressed in the context of palliative care (Figure 11).

Living with a physical disability

Living with a physical disability has presented ongoing challenges for Chaikhwa, particularly in a profession that demands physical strength and mobility. However, she has embraced her new reality with grace, adapting her nursing practice to accommodate her limitations. Chaikhwa has become an inspiration to others, showing that it is possible to continue pursuing one’s passions despite physical challenges.

She emphasizes the importance of resilience and positive thinking, urging others to look beyond their difficulties and focus on the opportunities that challenges present. Her message is clear: despite the obstacles, everyone has a purpose in life, and it is possible to create positive change, not only for oneself but also for future generations.

## Conclusions

Chaikhwa Nani Nelson’s journey from a small village in Botswana to a pioneering leader in oncology nursing is a powerful testament to the strength of the human spirit. Her story is one of overcoming immense personal challenges, using those experiences to drive positive change in her community, and achieving recognition on an international stage. Through her work, Chaikhwa has not only improved the lives of cancer patients but has also set a precedent for future healthcare professionals in Botswana. Her contributions to oncology, her advocacy for patient-centered care, and her leadership in establishing cancer support systems in Botswana will continue to have a lasting impact on healthcare.
